# Prediction of Antidepressant Treatment Response and Remission Using an Ensemble Machine Learning Framework

**DOI:** 10.3390/ph13100305

**Published:** 2020-10-13

**Authors:** Eugene Lin, Po-Hsiu Kuo, Yu-Li Liu, Younger W.-Y. Yu, Albert C. Yang, Shih-Jen Tsai

**Affiliations:** 1 Department of Biostatistics, University of Washington, Seattle, WA 98195, USA; 2 Department of Electrical & Computer Engineering, University of Washington, Seattle, WA 98195, USA; 3 Graduate Institute of Biomedical Sciences, China Medical University, Taichung 40402, Taiwan; 4 Department of Public Health, Institute of Epidemiology and Preventive Medicine, National Taiwan University, Taipei 10617, Taiwan; phkuo@ntu.edu.tw; 5 Center for Neuropsychiatric Research, National Health Research Institutes, Miaoli County 35053, Taiwan; ylliou@nhri.org.tw; 6 Yu’s Psychiatric Clinic, Kaohsiung 802211, Taiwan; yuwuyang@ms27.hinet.net; 7 Division of Interdisciplinary Medicine and Biotechnology, Beth Israel Deaconess Medical Center/Harvard Medical School, Boston, MA 02215, USA; accyang@gmail.com; 8 Institute of Brain Science, National Yang-Ming University, Taipei 112304, Taiwan; 9 Department of Psychiatry, Taipei Veterans General Hospital, Taipei 11217, Taiwan; 10 Division of Psychiatry, National Yang-Ming University, Taipei 112304, Taiwan

**Keywords:** antidepressant, ensemble learning, feature selection, machine learning, major depressive disorder, pharmacogenomics, single nucleotide polymorphisms

## Abstract

In the wake of recent advances in machine learning research, the study of pharmacogenomics using predictive algorithms serves as a new paradigmatic application. In this work, our goal was to explore an ensemble machine learning approach which aims to predict probable antidepressant treatment response and remission in major depressive disorder (MDD). To discover the status of antidepressant treatments, we established an ensemble predictive model with a feature selection algorithm resulting from the analysis of genetic variants and clinical variables of 421 patients who were treated with selective serotonin reuptake inhibitors. We also compared our ensemble machine learning framework with other state-of-the-art models including multi-layer feedforward neural networks (MFNNs), logistic regression, support vector machine, C4.5 decision tree, naïve Bayes, and random forests. Our data revealed that the ensemble predictive algorithm with feature selection (using fewer biomarkers) performed comparably to other predictive algorithms (such as MFNNs and logistic regression) to derive the perplexing relationship between biomarkers and the status of antidepressant treatments. Our study demonstrates that the ensemble machine learning framework may present a useful technique to create bioinformatics tools for discriminating non-responders from responders prior to antidepressant treatments.

## 1. Introduction

Nowadays researchers have been making significant progress in the interdisciplinary fields of pharmacogenomics, machine learning, and psychiatry [1,2,3]. In the arena of pharmacogenomics, the goal of machine learning research is to provide predictive algorithms that can in general help facilitate the investigation of how genetic variants and clinical variables can influence an individual’s treatment outcomes to drugs [1,2,3]. Latest advancements in machine learning algorithms have demonstrated their promising potentials with respect to pharmacogenomics for patients with psychiatric disorders [1,2,3]. For instance, machine learning approaches such as multi-layer feedforward neural networks (MFNNs) have been utilized to infer clinical treatment outcomes in patients with major depressive disorder (MDD) treated with antidepressants by using clinical characteristics and genetic variants such as single nucleotide polymorphisms (SNPs) [4]. Therefore, it has been suggested that machine learning and predictive algorithms play a key role in the future of pharmacogenomics since their relevant applications encompass various aspects of pharmacogenomics [5,6,7].

The use of machine learning and predictive algorithms in terms of predicting antidepressant treatment outcomes has been a focus of attention in pharmacogenomics research. For example, Kautzky et al. [8] used a random forests approach to correctly identify 25% of responders in patients with treatment-resistant depression by using a clinical variable (that is, melancholia) and 3 SNPs (including *BDNF* rs6265, *PPP3CC* rs7430, and *HTR2A* rs6313). In addition, Patel et al. [9] employed an alternating decision tree approach to predict treatment outcomes with 89% accuracy in patients with late-life depression by using structural imaging data and clinical variables such as age and mini-mental status examination scores. Furthermore, Chekroud et al. [10] proposed a gradient boosting machine learning approach which was able to estimate clinical remission with 59% accuracy in MDD patients by using 25 variables (such as Hamilton Rating Scale for Depression (HRSD)). Moreover, Iniesta et al. [11] utilized a regularized regression approach to forecast antidepressant treatment outcomes with clinically meaningful accuracy by using clinical and demographical variables. Maciukiewicz et al. [12] also demonstrated a support vector machine (SVM) approach to predict antidepressant treatment response with 52% accuracy in MDD patients by using SNPs. Finally, a recent study by Lin et al. [4] reported that an MFNN approach can foresee antidepressant treatment response (area under the receiver operating characteristic curve (AUC) = 0.8228) and remission (AUC = 0.8060) by using 10 SNPs and 6 clinical variables.

In a previous study, Lin et al. [4] proposed that the MFNN model can mediate the relationship between biomarkers and the responsiveness of antidepressant treatment in MDD patients. Here, we employed the same cohort of MDD patients and performed the first study on the prediction of antidepressant treatment response and remission with 10 genetic variants and 6 clinical variables by using a boosting ensemble algorithm in the Taiwanese population. Moreover, in order to foresee treatment outcomes, we utilized the wrapper-based feature selection algorithm [13] to carry out a small subset of suitable features from 10 genetic variants and 6 clinical variables. To the best of our knowledge, no previous studies have been conducted to assess predictive algorithms for antidepressant treatment outcomes in MDD patients by using boosting ensemble techniques with the wrapper-based feature selection algorithm. We chose the boosting ensemble algorithm as this algorithm is routinely used to deal with complicated applications in predictive modeling and may possess numerous advantages such as better prediction, greater consistency, robust generalization, and the prevention of overfitting [14,15]. The present study precisely compared the performance of the boosting ensemble algorithm to extensively-used machine learning models, including MFNNs, logistic regression, SVM, C4.5 decision tree, naïve Bayes, and random forests. Our analysis indicated that our boosting ensemble framework with feature selection (using fewer biomarkers) led to comparable performance.

## 2. Results

### 2.1. The Study Cohort in the Taiwanese Population

In brief, there were 421 patients with MDD, including 257 antidepressant treatment responders and 164 treatment non-responders. In addition, there were 139 remission patients and 282 non-remission patients. We also followed the previous study [4] and used six clinical variables in the subsequent analysis, including age at the time of the initial study, gender, 21-item HRSD at baseline, marital status, the number of previous depressive episodes (prior to the time of the initial study), and the status of suicide attempt history. In addition, demographic features (such as age, gender, and ethnicity) and their range of values for the 421 Taiwanese patients were detailed before [4].

### 2.2. Boosting Ensemble Model for Antidepressant Treatment Response

In this study, we followed the previous study [4] and used 16 biomarkers including 10 genetic variants and the aforementioned six clinical variables to build the predictive models for differentiating antidepressant treatment responders from non-responders by employing the boosting ensemble framework. The 10 genetic variants for antidepressant treatment response were detailed before [4], including *ABCA13* rs4917029, *BNIP3* rs9419139, *CACNA1E* rs704329, *EXOC4* rs6978272, *GRIN2B* rs7954376, *LHFPL3* rs4352778, *NELL1* rs2139423, *NUAK1* rs2956406, *PREX1* rs4810894, and *SLIT3* rs139863958 SNPs (see Methods).

First, for predicting antidepressant treatment response, we used the wrapper-based feature selection algorithm (see Methods) to identify 15 features (including 10 SNPs and 5 clinical variables) from the aforementioned 16 biomarkers. The selected five clinical variables encompass age at the time of the initial study, gender, 21-item HRSD at baseline, the number of previous depressive episodes (prior to the time of the initial study), and the status of suicide attempt history. That is, the clinical variable of marital status was removed after applying the wrapper-based feature selection algorithm. In addition, marital status was not linked to antidepressant treatment response (*p* = 0.107) based on the chi-square test.

For the boosting ensemble model for forecasting antidepressant treatment response, we performed two different datasets using the selected 15 features as well as the original 16 biomarkers. As indicated in Table 1, the average value of AUC for the boosting ensemble prediction model with the wrapper-based feature selection algorithm was 0.8265 (standard deviation = 0.0574) by using the selected 15 features.

On the other hand, for forecasting antidepressant treatment response, the average value of AUC for the boosting ensemble prediction model without feature selection was 0.8236 (standard deviation = 0.0564) by using the original 16 biomarkers (Table 1). Although the average values of AUC, sensitivity, and specificity for the boosting ensemble prediction model with the wrapper-based feature selection algorithm were higher than the ones for the boosting ensemble prediction model without feature selection, there was no statistically significant difference between these two average AUC (*p* = 0.719), sensitivity (*p* = 0.109), and specificity (*p* = 0.915) values as shown in Table S1 in the Supplementary file. Therefore, the boosting ensemble prediction model with the wrapper-based feature selection algorithm (using 15 biomarkers) gave comparable performance as the one without feature selection (using 16 biomarkers) in terms of AUC, sensitivity, and specificity for antidepressant treatment response.

### 2.3. Benchmarking for Antidepressant Treatment Response

To evaluate the performance of our approach for predictive models for antidepressant treatment response, we compared the boosting ensemble model with other state-of-the-art methods, including logistic regression, SVM, C4.5 decision tree, naïve Bayes, and random forests (Table 1). In addition, the average value of AUC for the MFNN model was 0.8228 ± 0.0571 in terms of predicting antidepressant treatment response [4].

After comparison, the boosting ensemble model with the wrapper-based feature selection algorithm had the maximal AUC in all cases for antidepressant treatment response. The best AUC was 0.8265 ± 0.0574, which was based on the boosting ensemble model with the wrapper-based feature selection algorithm (Table 1). Our analysis indicated that the boosting ensemble model with the wrapper-based feature selection algorithm was well-suited for predictive models for antidepressant treatment response.

However, there was no statistically significant difference in the average AUC, sensitivity, and specificity values between logistic regression and the boosting ensemble model with the wrapper-based feature selection algorithm (*p* = 0.225, 0.064, and 0.653, respectively) (Table S1). Similarly, there was no statistically significant difference in the average AUC, sensitivity, and specificity values between the MFNN model and the boosting ensemble model with the wrapper-based feature selection algorithm (*p* = 0.648, 0.215, and 0.069, respectively) (Table S1). Therefore, the boosting ensemble prediction model with the wrapper-based feature selection algorithm (using 15 biomarkers) gave comparable performance as logistic regression and the MFNN model (using 16 biomarkers) in terms of AUC, sensitivity, and specificity for antidepressant treatment response.

### 2.4. Boosting Ensemble Model for Antidepressant Remission

Moreover, we employed 16 biomarkers including 10 genetic variants and the aforementioned 6 clinical variables to build the predictive models for differentiating antidepressant remission from non-remission by employing the boosting ensemble framework. The 10 genetic variants for remission encompass *ARNTL* rs11022778, *CAMK1D* rs2724812, *GABRB3* rs12904459, *GRM8* rs35864549, *NAALADL2* rs9878985, *NCALD* rs483986, *PLA2G4A* rs12046378, *PROK2* rs73103153, *RBFOX1* rs17134927, and *ZNF536* rs77554113 SNPs (see Methods) [4].

To foresee antidepressant remission, we utilized the wrapper-based feature selection algorithm (see Methods) to identify 15 features (including 10 SNPs and five clinical variables) from the aforementioned 16 biomarkers for remission. The selected 5 clinical variables include age at the time of the initial study, gender, 21-item HRSD at baseline, the number of previous depressive episodes (prior to the time of the initial study), and the status of suicide attempt history. Namely, the clinical variable of marital status was removed after applying the wrapper-based feature selection algorithm. In addition, marital status was not linked to antidepressant remission (*p* = 0.898) based on the chi-square test. 

For the boosting ensemble model for forecasting antidepressant remission, we conducted two different datasets using the selected 15 features as well as the original 16 biomarkers. As shown in Table 2, the average value of AUC for the boosting ensemble prediction model with the wrapper-based feature selection algorithm was 0.8122 (standard deviation = 0.0702) by using the selected 15 features.

On the other hand, for forecasting antidepressant remission, the average value of AUC for the boosting ensemble prediction model without feature selection was 0.8111 (standard deviation = 0.0691) by using the original 16 biomarkers (Table 2). Although the average values of AUC, sensitivity, and specificity for the boosting ensemble prediction model with the wrapper-based feature selection algorithm were higher than the ones for the boosting ensemble prediction model without feature selection, there was no statistically significant difference between these two average AUC (*p* = 0.911), sensitivity (*p* = 0.641), and specificity (*p* = 0.628) values as shown in Table S2 in the Supplementary file. Thus, the boosting ensemble prediction model with the wrapper-based feature selection algorithm (using 15 biomarkers) obtained comparable performance as the one without feature selection (using 16 biomarkers) in terms of AUC for antidepressant remission.

### 2.5. Benchmarking for Antidepressant Remission

To evaluate the performance of our approach for predictive models for antidepressant remission, we compared the boosting ensemble model with other state-of-the-art methods, including logistic regression, SVM, C4.5 decision tree, naïve Bayes, and random forests (Table 2). In addition, the average value of AUC for the MFNN model was 0.8060 ± 0.0722 in terms of predicting antidepressant remission [4].

After comparison, the boosting ensemble model with the wrapper-based feature selection algorithm had the maximal AUC in all cases. The best AUC was 0.8111 ± 0.0691, which was based on the boosting ensemble model with the wrapper-based feature selection algorithm (Table 2). Our analysis suggested that the boosting ensemble model with the wrapper-based feature selection algorithm was well-suited for predictive models for antidepressant remission.

However, there was no statistically significant difference in the average AUC, sensitivity, and specificity values between logistic regression and the boosting ensemble model with the wrapper-based feature selection algorithm (*p* = 0.191, 0.330, and 0.200, respectively) (Table S2). Similarly, there was no statistically significant difference in the average AUC, sensitivity, and specificity values between the MFNN model and the boosting ensemble model with the wrapper-based feature selection algorithm (*p* = 0.539, 0.365, and 0.781, respectively) (Table S2). Therefore, the boosting ensemble prediction model with the wrapper-based feature selection algorithm (using 15 biomarkers) gave comparable performance as logistic regression and the MFNN model (using 16 biomarkers) in terms of AUC, sensitivity, and specificity for antidepressant remission.

## 3. Discussion

To our knowledge, this is the first study to date to leverage a boosting ensemble predictive framework with the wrapper-based feature selection algorithm for building predictive models of antidepressant treatment response and antidepressant remission among Taiwanese patients with MDD. Our analysis found that the boosting ensemble predictive framework with the wrapper-based feature selection algorithm (using 15 biomarkers) performed comparably to other state-of-the-art predictive models (using 16 biomarkers) such as logistic regression and the MFNN model in terms of AUC for distinguishing antidepressant treatment non-responders from responders in MDD. We also pinpointed that the boosting ensemble predictive framework with the wrapper-based feature selection algorithm (using 15 biomarkers) achieved comparatively to other state-of-the-art predictive models (using 16 biomarkers) such as logistic regression and the MFNN model in terms of AUC for forecasting antidepressant remission in MDD. In addition, we identified five common clinical biomarkers for predicting both antidepressant treatment response and antidepressant remission by using the wrapper-based feature selection algorithm, namely age at the time of the initial study, gender, 21-item HRSD at baseline, the number of previous depressive episodes, and the status of suicide attempt history. By leveraging the SNP data and clinical variables, we establish the predictive models of antidepressant treatment response and antidepressant remission by using the boosting ensemble predictive framework with the wrapper-based feature selection algorithm. Our results also implicate that our boosting ensemble predictive framework with feature selection may serve as an applicable bioinformatics tool for performing predictive models for foreseeing antidepressant treatment response as well as antidepressant remission with clinically meaningful accuracy. Accordingly, our boosting ensemble predictive framework with feature selection is a proof of concept of machine learning predictive approaches for drug efficacy prior to antidepressant therapy in MDD.

By using the wrapper-based feature selection algorithm, we eliminated one clinical variable, namely marital status. We also verified that marital status was not linked to antidepressant treatment response or antidepressant remission by using the chi-square test. In accordance with our study, no effect of marital status was observed in several previous studies [16,17]. On the other hand, it has been reported that married patients with MDD responded better to antidepressants as compared to unmarried ones [18]. Possible explanations for the discrepancies regarding marital status may be due to inadequate sample sizes, distinct study designs, diverse populations, and various confounding effects [1,19].

Furthermore, it is worthwhile to bring the discussion on the similarity of the methods for carrying out the prediction of antidepressant treatment response or antidepressant remission in MDD patients in our study. We observed that the boosting ensemble model with the wrapper-based feature selection algorithm (using 15 biomarkers), the boosting ensemble model (using 16 biomarkers), logistic regression (using 16 biomarkers), and the MFNN model (using 16 biomarkers) were equivalent methods from a statistics standpoint. That is, these four models had similar predictive power. First, it may be advantageous to use the boosting ensemble model with the wrapper-based feature selection algorithm because fewer biomarkers are required to achieve comparable performance, especially when the lack of data occurs (for example, missing data such as marital status in this study) [13,20,21]. Second, the logistic regression model generally serves as a basis for the benchmarking task [4]. Thus, it was encouraging that our proposed boosting ensemble framework with fewer biomarkers was comparable to the logistic regression model.

## 4. Materials and Methods

### 4.1. Study Population

The study subjects were mainly original to a previous study by Lin et al. [4]. Briefly, the study cohort consists of 455 patients with MDD who were treated with selective serotonin reuptake inhibitors (SSRIs), where the subjects were part of the International SSRI Pharmacogenomics Consortium project [4]. The study cohort was further reduced to 421 patients after quality control procedures (see Section 4.3). Patients were assessed by board-certified psychiatrists regularly at baseline and week 2, 4, and 8 using the 21-item HRSD [4]. Experiments were conducted in accordance with the Declaration of Helsinki and approved by the Institutional Review Board of Taipei Veterans General Hospital (VGHIRB No.: 2014-06-001B). Written informed consents were obtained from all participants ensuring adequate understanding of the study.

### 4.2. Measurement

Measurements of treatment response and remission were obtained for participants as follows [4,22]. First, we measured the percentage change of HRSD (that is, %ΔHRSD) and classified the participant as “non-responder” if the percentage change was greater than −50%; otherwise, we classified the participant as “responder” [4]. Second, we measured the sum score of 21-item HRSD at the 8th week of antidepressant treatment and classified the participant as “non-remission” if the sum score was greater than 7; otherwise, we classified the participant as “remission” [4].

### 4.3. Genotyping Dat and Quality Controls

For all subjects, we performed SNP genotyping by using Illumina HumanOmniExpressExome BeadChips in the International SSRI Pharmacogenomics Consortium [4,23]. In addition, we carried out quality control procedures (such as kinship, sample quality, and population stratification) and then removed a total of 34 subjects [4]. As a result, we retained 421 MDD patients for the subsequent analysis. 

### 4.4. Key SNPs

The key SNPs were detailed in the previous study by Lin et al. [4]. In brief, for predictive modeling in antidepressant treatment response, we selected 10 top-rated SNPs showing an evidence of association with antidepressant treatment response using a significant level of *p* < 7.5 × 10^−5^ in an odds ratio analysis [4]. These 10 key SNPs encompass rs4917029 adjacent to the *ABCA13* gene, rs9419139 adjacent to the *BNIP3* gene, rs704329 in the *CACNA1E* gene, rs6978272 in the *EXOC4* gene, rs7954376 adjacent to the *GRIN2B* gene, rs4352778 in the *LHFPL3* gene, rs2139423 in the *NELL1* gene, rs2956406 in the *NUAK1* gene, rs4810894 adjacent to the *PREX1* gene, and rs139863958 adjacent to the *SLIT3* gene [4].

In addition, for predictive modeling in antidepressant remission, we identified 10 top-rated SNPs showing an evidence of association with antidepressant remission using a significant level of *p* < 9.9 × 10^−5^ in an odds ratio analysis [4]. These 10 key SNPs encompass rs11022778 in the *ARNTL* gene, rs2724812 in the *CAMK1D* gene, rs12904459 adjacent to the *GABRB3* gene, rs35864549 adjacent to the *GRM8* gene, rs9878985 in the *NAALADL2* gene, rs483986 in the *NCALD* gene, rs12046378 adjacent to the *PLA2G4A* gene, rs73103153 adjacent to the *PROK2* gene, rs17134927 in the *RBFOX1* gene, and rs77554113 adjacent to the *ZNF536* gene [4].

### 4.5. Wrapper-Based Feature Selection Algorithm

In this study, we employed the wrapper-based feature selection algorithm [13], where the feature selection algorithm acts as a wrapper around the predictive algorithm. The wrapper-based method performs the best-first search for a good subset of features by using the predictive algorithm itself as part of the procedure for assessing feature subsets [13,20]. The best-first search starts with an empty set of features and then searches forward to choose a potential subset of features by a greedy hill-climbing approach augmented with a backtracking technique [13,20].

### 4.6. Boosting Ensemble Predictive Framework

**Figure 1:**
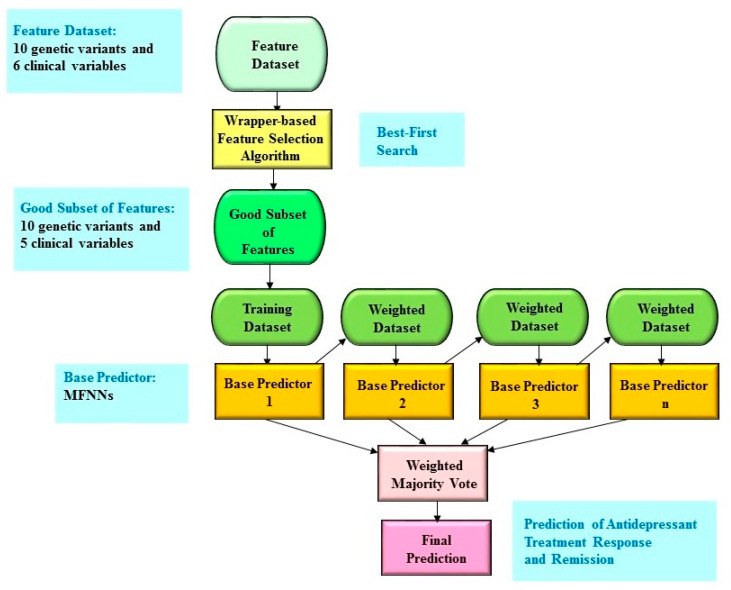
The schematic illustration of the boosting ensemble predictive framework with feature selection. First, the wrapper-based feature selection algorithm is performed to select a good subset of features, which provides the input to the boosting ensemble predictive algorithm. Next, the idea of the boosting ensemble predictive algorithm is to train base predictors in a sequential process such that each base predictor manages to adjust its predecessor. A higher weight is then designated to samples that are wrongly predicted by earlier rounds. In other words, base predictors are performed sequentially by using a weighted version of the dataset within the training phase. Finally, the prediction is generated by a weighted majority vote. In this study, we chose multi-layer feedforward neural networks (MFNNs) as the base predictor.

In this study, we integrated a boosting ensemble model with the wrapper-based feature selection algorithm. Figure 1 shows the illustrative diagram of the boosting ensemble predictive framework which is combined with the wrapper-based feature selection algorithm. More specifically, we utilized a boosting ensemble model called LogitBoost [24] and employed the Waikato Environment for Knowledge Analysis (WEKA) software (which is available from https://www.cs.waikato.ac.nz/ml/weka/) [25] to conduct the boosting ensemble predictive framework. All the experiments were conducted on a computer with Intel (R) Core (TM) i5-4210U, 4 GB RAM, and Windows 7.

The LogitBoost algorithm is a boosting ensemble model, which incorporates the performance of many weak predictive models (also referred to as base predictive models) to accomplish a robust predictive model with higher accuracy [26]. Moreover, the LogitBoost algorithm employs a binomial log-likelihood algorithm that adjusts the predictive error linearly, thereby tending to be robust in handling outliers and noisy data [26]. The base predictive model we utilized is an MFNN, which consists of one input layer, one hidden layer, and one output layer. Here, for the LogitBoost algorithm, we used the default parameters of WEKA, such as 1.0 for the shrinkage parameter, 100 for the batch size, 3.0 for the Z max threshold, and 10 for the number of iterations. In addition, for the MFNN model, WEKA’s parameters were chosen as follows: the momentum = 0.01, the learning rate = 0.001 or 0.002, and the batch size = 100 [4,26]. The momentum, learning rate, and batch size were set at the given values using a grid search approach [27].

### 4.7. Machine Learning Algorithms for Benchmarking

For the benchmarking task in the present study, we employed five state-of-the-art machine learning algorithms including logistic regression, SVM, C4.5 decision tree, naïve Bayes, and random forests to compare with the boosting ensemble predictive framework. We performed the analyses for these five machine learning algorithms using the WEKA software [25] and a computer with Intel (R) Core (TM) i5-4210U, 4 GB RAM, and Windows 7.

As a basis for comparison, the logistic regression model is the standard approach for predictive modeling in clinical applications [4].

The SVM model [28] utilizes a kernel function to map the training vectors into a higher dimensional space and then identifies a linear separating hyperplane with the maximal margin [29]. In this study, we used the polynomial kernel with the exponent value of 1.0.

The C4.5 decision tree model constructs decision trees in top-down processing and prunes the decision trees using the notion of information entropy [20]. Here, we used the default parameters of WEKA, such as 0.25 for the confidence factor and two for the minimum number of instances per leaf node.

The naïve Bayes model measures the probability that a given instance belongs to a certain class (for example, “non-responder” or “responder” in this study) by using the Bayes’ theorem [26]. Here, we used the default parameters of WEKA for the naïve Bayes model, such as 100 for the batch size.

The random forests model builds a collection of decision trees during training and then produces the class that is the mode of the classes among the individual trees [30]. Here, we used the default parameters of WEKA for the random forests model; for example, 100 for the batch size and 100 for the number of iterations.

### 4.8. Evaluation of the Predictive Performance

In this study, we utilized the receiver operating characteristic (ROC) methodology and determined the AUC to assess the performance of predictive models [20,29,31]. The better the prediction model, the higher the AUC [20,31]. In addition, we calculated sensitivity (namely, the proportion of correctly predicted responders of all tested responders) as

Sensitivity = True Positive/(True Positive + False Negative)

and specificity (namely, the proportion of correctly predicted non-responders of all the tested non-responders) as

Specificity = True Negative/(True Negative + False Positive)

Furthermore, we performed the repeated 10-fold cross-validation method to examine the generalization of predictive models [20,32]. In brief, the whole dataset was randomly split into ten separate segments. The predictive model used nine-tenths of the dataset for training and the remaining tenth of the dataset for testing. Then, the previous step was repeated nine more times by using distinct nine-tenths of the dataset for training and a distinct tenth of the dataset for testing. Finally, the data are presented as mean ± standard deviation.

The Student’s *t* test was conducted to measure the difference in the means of two continuous variables (for example, AUC, sensitivity, and specificity) [4].

## 5. Conclusions

In conclusion, we proposed a boosting ensemble predictive framework with the wrapper-based feature selection algorithm for predicting antidepressant treatment response and remission in Taiwanese patients with MDD. The present results suggest that our boosting ensemble predictive framework with the wrapper-based feature selection algorithm may leverage a feasible way to create predictive algorithms for forecasting antidepressant treatment response and remission with clinically meaningful accuracy. Furthermore, we revealed the similarity of the machine learning methods in terms of predictive performance when we compared the boosting ensemble predictive framework (with fewer biomarkers) to other state-of-the-art models such as MFNNs and logistic regression. In future work, we will further explore the utility of the boosting ensemble predictive framework and investigate ways to demonstrate the superiority of new approaches. Therefore, we could assume that the analysis of the present study might be generalized for machine learning studies of pharmacogenomics in foreseeing treatment response and remission for human diseases. Moreover, the results would be utilized to build bioinformatics tools in pharmacogenomics within the next few years. All in all, it is crucial to explore further findings into the role of the boosting ensemble predictive framework examined in this study by using various independent samples of replication studies.

## Figures and Tables

**Table 1:** The results of repeated 10-fold cross-validation experiments for predicting treatment response with genetic variants and clinical variables using the boosting ensemble model with feature selection, the boosting ensemble model, logistic regression, SVM, C4.5 decision tree, naïve Bayes, and random forests.

Algorithm	AUC	Sensitivity	Specificity	Number of Biomarkers
Boosting ensemble with feature selection	0.8265 ± 0.0574	0.7651 ± 0.0574	0.7114 ± 0.0721	15
Boosting ensemble	0.8236 ± 0.0564	0.7517 ± 0.0602	0.7103 ± 0.0736	16
Logistic regression	0.8168 ± 0.0553	0.7493 ± 0.0626	0.7066 ± 0.0785	16
SVM	0.7306 ± 0.0685	0.7499 ± 0.0624	0.7113 ± 0.0785	16
C4.5 decision tree	0.6802 ± 0.0853	0.6926 ± 0.0654	0.6468 ± 0.0762	16
Naïve Bayes	0.8176 ± 0.0593	0.7439 ± 0.0630	0.6844 ± 0.0771	16
Random forests	0.7852 ± 0.0644	0.7078 ± 0.0670	0.6234 ± 0.0786	16

AUC = the area under the receiver operating characteristic curve; SVM = support vector machine. The data are presented as mean ± standard deviation.

**Table 2:** The results of repeated 10-fold cross-validation experiments for predicting remission with genetic variants and clinical variables using the boosting ensemble model with feature selection, the boosting ensemble model, logistic regression, SVM, C4.5 decision tree, naïve Bayes, and random forests.

Algorithm	AUC	Sensitivity	Specificity	Number of Biomarkers
Boosting ensemble with feature selection	0.8122 ± 0.0702	0.7807 ± 0.0584	0.6589 ± 0.0872	15
Boosting ensemble	0.8111 ± 0.0691	0.7768 ± 0.0597	0.6529 ± 0.0875	16
Logistic regression	0.7985 ± 0.0772	0.7722 ± 0.0645	0.6753 ± 0.0932	16
SVM	0.7149 ± 0.0738	0.7679 ± 0.0624	0.6620 ± 0.0910	16
C4.5 decision tree	0.6276 ± 0.0955	0.6912 ± 0.0619	0.5409 ± 0.0852	16
Naïve Bayes	0.8078 ± 0.0709	0.7608 ± 0.0649	0.6743 ± 0.0869	16
Random forests	0.7733 ± 0.0766	0.7432 ± 0.0523	0.5735 ± 0.0826	16

AUC = the area under the receiver operating characteristic curve; SVM = support vector machine. The data are presented as mean ± standard deviation.

## Supplementary Materials

The following are available online at https://www.mdpi.com/1424-8247/13/10/305/s1, Table S1: The difference in the means of AUC, sensitivity, and specificity for predicting treatment response between the boosting ensemble model with feature selection and other models including the boosting ensemble model, logistic regression, SVM, C4.5 decision tree, naïve Bayes, random forests, and MFNN models, Table S2: The difference in the means of AUC, sensitivity, and specificity for predicting remission between the boosting ensemble model with feature selection and other models including the boosting ensemble model, logistic regression, SVM, C4.5 decision tree, naïve Bayes, random forests, and MFNN models.
