# Supplementary material for: Prediction of Antidepressant Treatment Response and Remission Using an Ensemble Machine Learning Framework
Source: Pharmaceuticals (Basel). 2020 Oct 13;13(10):305. doi: 10.3390/ph13100305 (PMC7599952; doi:10.3390/ph13100305)
Supplement: Supplementary file 1 [file pharmaceuticals-13-00305-s001.pdf]

pharmaceuticals

Article

Supplementary Materials for

Prediction of Antidepressant Treatment Response and Remission Using an Ensemble Machine Learning Framework

Eugene Lin [1,2,3,*], Po-Hsiu Kuo [4], Yu-Li Liu [5], Younger W-Y Yu [6], Albert C. Yang [7,8], and Shih-Jen Tsai [8,9,10,*]

[1] Department of Biostatistics, University of Washington, Seattle, WA 98195, USA
[2] Department of Electrical & Computer Engineering, University of Washington, Seattle, WA 98195, USA
[3] Graduate Institute of Biomedical Sciences, China Medical University, Taichung 40402, Taiwan
[4] Department of Public Health, Institute of Epidemiology and Preventive Medicine, National Taiwan University, Taipei 10617, Taiwan
[5] Center for Neuropsychiatric Research, National Health Research Institutes, Miaoli County 35053, Taiwan
[6] Yu's Psychiatric Clinic, Kaohsiung 802211, Taiwan
[7] Division of Interdisciplinary Medicine and Biotechnology, Beth Israel Deaconess Medical Center/Harvard Medical School, Boston, MA 02215, USA
[8] Institute of Brain Science, National Yang-Ming University, Taipei 112304, Taiwan
[9] Department of Psychiatry, Taipei Veterans General Hospital, Taipei 11217, Taiwan
[10] Division of Psychiatry, National Yang-Ming University, Taipei 112304, Taiwan
* Correspondence: lines@uw.edu (E. L.); tsai610913@gmail.com (S.-J. T.)

Table S1. The difference in the means of AUC, sensitivity, and specificity for predicting treatment response between the boosting ensemble model with feature selection and other models including the boosting ensemble model, logistic regression, SVM, C4.5 decision tree, naïve Bayes, random forests, and MFNN models.

Algorithms	P value for AUC	P value for Sensitivity	P value for Specificity
BEFS vs. Boosting ensemble	0.719	0.109	0.915
BEFS vs. Logistic regression	0.225	0.064	0.653
BEFS vs. SVM	< 0.0001	0.075	0.993
BEFS vs. C4.5 decision tree	< 0.0001	< 0.0001	< 0.0001
BEFS vs. Naïve Bayes	0.282	0.014	0.011
BEFS vs. Random forests	< 0.0001	< 0.0001	< 0.0001
BEFS vs. MFNN	0.648	0.215	0.069

AUC = the area under the receiver operating characteristic curve; BEFS = boosting ensemble model with feature selection; MFNN = multilayer feedforward neural network; SVM = support vector machine. The P value was obtained by the Student's t test.

Table S2. The difference in the means of AUC, sensitivity, and specificity for predicting remission between the boosting ensemble model with feature selection and other models including the boosting ensemble model, logistic regression, SVM, C4.5 decision tree, naïve Bayes, random forests, and MFNN models.

**Algorithms**	**P value for AUC**	**P value for Sensitivity**	**P value for Specificity**
BEFS vs. Boosting ensemble	0.911	0.641	0.628
BEFS vs. Logistic regression	0.191	0.330	0.200
BEFS vs. SVM	< 0.0001	0.136	0.806
BEFS vs. C4.5 decision tree	< 0.0001	< 0.0001	< 0.0001
BEFS vs. Naïve Bayes	0.660	0.024	0.212
BEFS vs. Random forests	0.0002	< 0.0001	< 0.0001
BEFS vs. MFNN	0.539	0.365	0.781

AUC = the area under the receiver operating characteristic curve; BEFS = boosting ensemble model with feature selection; MFNN = multilayer feedforward neural network; SVM = support vector machine. The P value was obtained by the Student's t test.

© 2020 by the authors. Submitted for possible open access publication under the terms and conditions of the Creative Commons Attribution (CC BY) license (http://creativecommons.org/licenses/by/4.0/).
